# Protein Dimerization via Tyr Residues: Highlight of a Slow Process with Co-Existence of Numerous Intermediates and Final Products

**DOI:** 10.3390/ijms23031174

**Published:** 2022-01-21

**Authors:** Anouchka Gatin, Patricia Duchambon, Guillaume van der Rest, Isabelle Billault, Cécile Sicard-Roselli

**Affiliations:** 1 Université Paris-Saclay, CNRS, Institut de Chimie Physique UMR 8000, CEDEX, 91405 Orsay, France; anouchka.gatin@universite-paris-saclay.fr (A.G.); guillaume.van-der-rest@universite-paris-saclay.fr (G.v.d.R.); isabelle.billault@universite-paris-saclay.fr (I.B.); 2 Université Paris-Saclay, CNRS, Institut Curie UMR 9187, INSERM U1196, CEDEX, 91405 Orsay, France; patricia.duchambon@curie.fr

**Keywords:** tyrosine dimers, proteins, oxidative stress, radicals

## Abstract

Protein dimerization via tyrosine residues is a crucial process in response to an oxidative attack, which has been identified in many ageing-related pathologies. Recently, it has been found that for isolated tyrosine amino acid, dimerization occurs through three types of tyrosine–tyrosine crosslinks and leads to at least four final products. Herein, considering two protected tyrosine residues, tyrosine-containing peptides and finally proteins, we investigate the dimerization behavior of tyrosine when embedded in a peptidic sequence. After azide radical oxidation and by combining UPLC-MS and H/D exchange analyzes, we were able to evidence: (i) the slow kinetics of Michael Addition Dimers (MAD) formation, i.e., more than 48 h; (ii) the co-existence of intermediates and final cyclized dimer products; and (iii) the probable involvement of amide functions to achieve Michael additions even in proteins. This raises the question of the possible in vivo existence of both intermediates and final entities as well as their toxicity and the potential consequences on protein structure and/or function.

## 1. Introduction

**Scheme 1:**
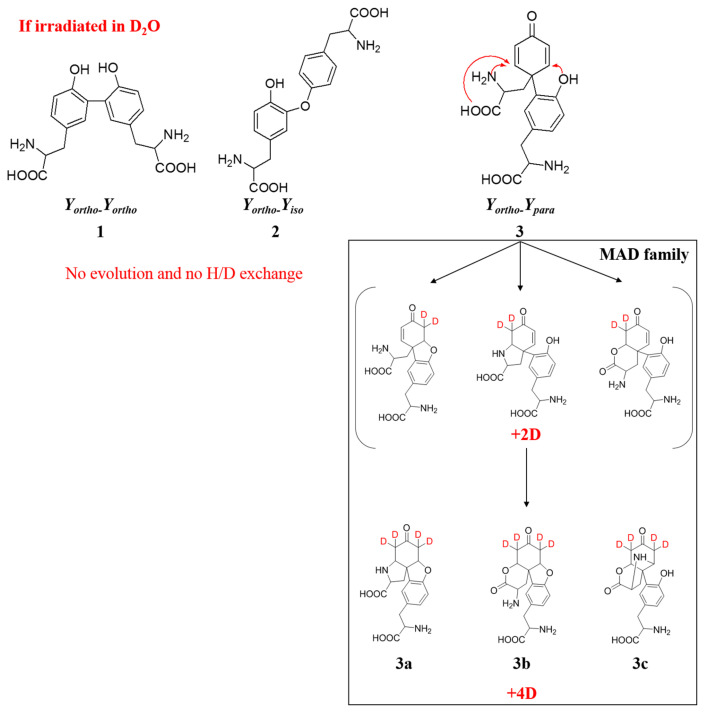
Representation of the tyrosine dimer cross-links and evolved structures composing the MAD family.

In vivo, radical production is a continuous process. It is necessary to trigger many biological responses. Nevertheless, its main side effect, called oxidative stress, is caused by a too high radical production leading to cellular dysfunctions [1,2,3,4,5]. Proteins are one of the main target of radicals and their oxidative modification can be linked to pathologies if not recognized early enough by a repair system or proteasome to be degraded [6,7]. For example, hydroxyl radical oxidation of tyrosine residues leads to diverse products, 3–4 dihydroxyphenylalanine formed by the addition of HO^•^ but also dimers after hydrogen abstraction [8,9]. Dityrosine covalent cross-link was identified as a marker of oxidative stress and its detection has been related with several pathologies [10,11]. Considering the high sensitivity needed to detect tyrosine dimers in tissues or cell extracts, the main detection methods are based on fluorescence or antibody response sensing [12,13]. In our previous studies [14,15], we could evidence new tyrosine dimeric structures formed after oxidative radical attack, among which only one is fluorescent. In these previous studies, the radicals were produced radiolytically. The hydroxyl radical was used to mimic in vivo conditions and the azide radical to focus only on the dimerization process. With a lower redox potential, the azide radical is more selective and induces only dimers, as it only reacts on the Tyr phenolic group. These studies also concluded that dimerization only originates from an initial oxygen-centered radical, which delocalizes to two mesomeric forms containing a carbon-centered radical characterized by absorption spectroscopy [16,17]. Their recombinations are at the origin of covalent bonds and we previously showed that three different cross-links, named 1, 2 and 3 in Scheme 1, are responsible for the dimers formed [15]. Whatever the target of oxidative radicals, an amino acid, a peptide or a protein, more than three Tyr dimeric structures were evidenced [14]. Taking into account the radical intermediates and combining MS analysis, H/D exchange and specific deuteration, we were able to propose five dimeric structures summarized in Scheme 1 for isolated tyrosine [15]. We confirmed the presence of the two already known dimers, *ortho-ortho* (**1**) and *iso* (**2**) dityrosine [18,19,20], though three new dimeric structures (**3a**, **3b** and **3c**) were proposed for two additional products detected. These new products were named the Michael Addition Dimers (MAD) family as they all arise from two successive Michael additions [15]. On one side, the phenolic group can form a five-member ring and on the other side, either the amino or the carboxylic function can add to the α,β-unsaturated ketone system as depicted by the arrows in Scheme 1. Therefore, the MAD family is made of three intermediate species bearing one cyclization, and final products (**3a**, **3b** and **3c**) obtained after a second cyclization.

To transpose these dimers formation to a larger scale than the isolated amino acid, we then focused on a protein, human centrin 2 (HsCen2), containing a single COOH-terminal tyrosine that was shown to form di-tyrosine oligomers [21,22]. In that case, five new dimers were detected, which could be in agreement with some of the proposed structures [14]. Nevertheless, if one considered incorporation of the tyrosine residue within a full protein sequence, as opposed to the C-terminal residue, we could expect that this would prevent some Michael additions and thus reduce the number of dimers. 

Based on the new methodology, we developed a way to differentiate tyrosine dimers after oxidation [15]. The goal of this study is to enable a more precise knowledge of tyrosine oxidative dimerization in any type of systems from modified amino acids to proteins. H/D exchange with the solvent appeared as a reliable tool to discriminate the *ortho-ortho* and *iso* dimers from the MAD family. Actually, while no deuterium incorporation is possible for the *ortho-ortho* and *iso* dimers when irradiations are performed in D_2_O, cyclizations of the other species allow the incorporation of deuterium atoms [15]. More precisely, as depicted in Scheme 1, the MAD family is made of intermediate compounds formed after a first cyclization able to incorporate up to 2 D after H/D exchange of the ketone α hydrogens. Final products are generated after a second cyclization allowing a mass increase representing the incorporation of up to 4 D. Thus, MAD can be identified as intermediate or final species considering their mass increase after H/D exchange. In the case of tyrosine, only final dimers were detected [15]. In this work we applied this discriminating procedure to different modified tyrosines, tyrosine-containing peptides and finally proteins to have a better understanding of their dimerization behavior. For this aim, we first focused on a tyrosine protected on the amino side N-acetyl-L-Tyrosine (Ac-Y), a 5-amino acid peptide containing a C-terminal tyrosine (KTSLY) and compared their behavior with that of human centrin 2 as KTSLY is the C-terminal sequence of HsCen 2. Then, we considered tyrosine protected both on the amino and the carboxylic functions: N-acetyl-L-tyrosine ethyl ester (Ac-Y-Et) as the simplest model, the KTSLY peptide with an additional glycine C-terminal residue (KTSLYG) and finally, we looked at the diversity of detected dimers for those obtained for human calmodulin (CaM), a calciprotein containing two tyrosine residues in the middle of its sequence.

## 2. Results

### 2.1. Impact of the Amine Function Modification

#### 2.1.1. N-Acetyl-L-Tyrosine (Ac-Y)

**Figure 1:**
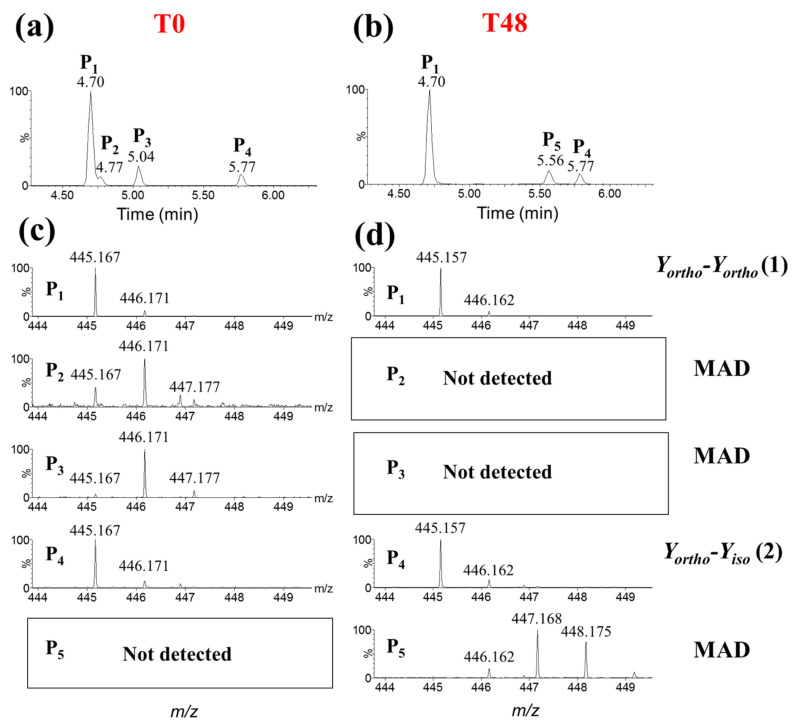
Oxidation of N-acetyl-L-tyrosine (Ac-Y) at 40 Gy in N_3_^•^ production conditions. Extracted ion chromatograms *m*/*z* 445.16 ± 0.05 ([M + H]^+^) after oxidation in H_2_O at (**a**) T0 (0 h); (**b**) T48 (48 h). Full scan MS spectra extracted at RT of dimers after oxidation in D_2_O at (**c**) T0 (0 h) and (**d**) T48 (48 h). Minor peaks in mass spectra arise from isotopic profiles.

In order to decipher the role of the amine function of isolated tyrosine, we selected Ac-Y that was irradiated to generate dimers. The sample was submitted to a gamma ray dose of 40 Gy under N_2_O atmosphere, which corresponds to a cumulated dose of 2 µM of azide radicals. Figure 1 represents the chromatograms of Ac-Y that was frozen just after irradiation called T0, for an ion extracted at *m*/*z* 445.16 corresponding to the expected covalent dimer i.e., [M + H]^+^ species (Table S1). From this chromatogram, we can clearly see the formation of four products (P_1_, P_2_, P_3_ and P_4_) with retention time (RT) between 4.5 and 6 min. Mass spectrum of each new species confirms that they all correspond to covalent dimers (Figure S1). After 48 h incubation at 37 °C (T48), P_2_ and P_3_ are not detected anymore and a new dimer, P_5_, is now detected at RT = 5.56 min. 

Figure 1c,d exhibits the modifications induced by performing the irradiations in D_2_O. First, either for T0 or T48, peaks P_1_ and P_4_ are the same as in H_2_O, with no mass spectra modification. Thus, they can be identified as the *Y_ortho_-Y_ortho_* (**1**) and *Y_ortho_-Y_iso_* (**2**) dimers. Considering that as for tyrosine, retention time of **1** is lower than that of **2** due to a more hydrophilic character of the dimer with two phenolic functions, we can assign P_1_ at 4.7 min to the *ortho-ortho* dimer and P_4_ at 5.7 min to the *iso*-dimer. In addition, it should be noticed that under these conditions, which include chromatographic separation in a H_2_O mobile phase, all the fast-exchangeable hydrogens, including the amide hydrogens are back-exchanged with the solvent to the hydrogen form. In D_2_O, P_2_ and P_3_ are only detected at T0 with a mass increase of about 1 (See Table S2 for precise values). As already stated, the deuterium incorporation is a tell-tale of cycloaddition. A single cycloaddition can lead to the incorporation of at most 2 D, and a double cycloaddition to at most 4 D (Scheme 1). After 48 h, P_2_ and P_3_ are not detected anymore, which indicates that they correspond to intermediate dimeric forms. Furthermore, for P_5_ incorporation of more than 2 deuterium atoms is observed, which shows that P_5_ has the possibility to incorporate more D atoms than P_2_ and P_3_, indicating that P_2_ and P_3_ are likely to be single cycloaddition products and P_5_ the final double cycloaddition product. This corroborates the fact that, after formation of the *ortho-para* cross-link (Scheme 1), dimeric species evolve leading to double cyclization, one with the phenol function and the second with most probably the carboxylate function as for Ac-Y the NH_2_ function is involved in an amide bond. Additionally, we evidence here that evolution to final dimeric structures is slower than that of free tyrosine [15]. 

#### 2.1.2. KTSLY Peptide

**Figure 2:**
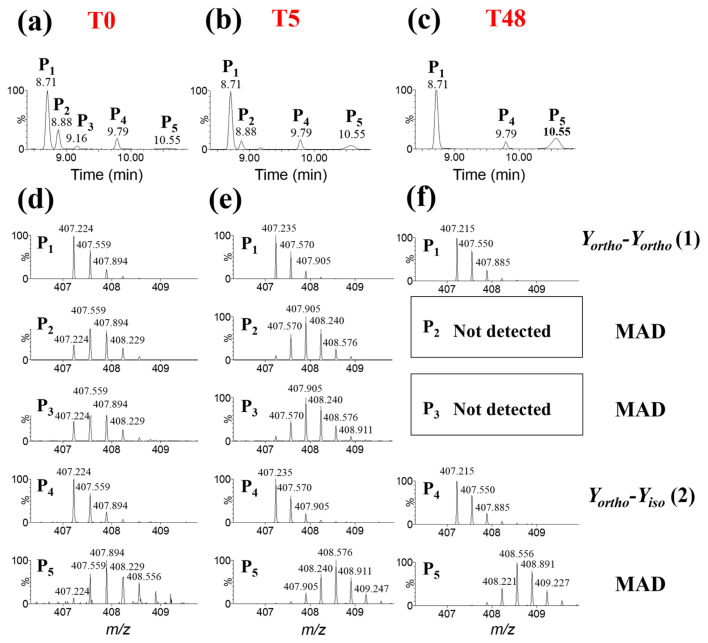
Oxidation of peptide KTSLY at 40 Gy in N_3_^•^ production conditions. Extracted ion chromatograms at *m*/*z* 407.22 ± 0.05 for [M + 3H]^3+^ after oxidation in H_2_O at (**a**) T0 (0 h), (**b**) T5 (5 h) and (**c**) T48 (48 h). Full scan MS spectra for [M + 3H]^3+^ extracted at RT of dimers after oxidation in D_2_O at (**d**) T0 (0 h), (**e**) T5 (5 h) and (**f**) T48 (48 h). Minor peaks in mass spectra arise from isotopic profiles.

As a second step, we applied the same methodology to a more complex system i.e., a 5 amino acid peptide KTSLY. In Gatin et al. [14], we already evidenced the formation of five dimers for this peptide but in this earlier study, time and temperature between irradiation and analysis were not strictly assessed. Here, Figure 2 illustrates the evolution of the number of chromatographic peaks corresponding to ion extracted at *m*/*z* 407.22, which corresponds to [M + 3H]^3+^ for covalent dimers KTSLY-^C^YLSTK^N^. At T0, four peaks are well-separated by LC–MS with intensities evolving with incubation at 37 °C. Previous MSMS experiments confirmed that they arise from Tyr dimerization [14].

H/D exchange experiments show that P_1_ and P_4_, present at T0 and T48, are not sensitive to the presence of deuterium atoms in the mixture (Figure 2b), thus they correspond to the *ortho-ortho* and *iso* dimer, respectively, as P_1_ appears more hydrophilic than P_4_. P_2_ and P_3_ show incorporation of less than two deuterium atoms from D_2_O at both T0 and T5 (Table S2), confirming that they correspond to a first cyclization and they evolve with time, most probably to the benefit of P_5_ obtained after a second cyclization, as it exhibits up to nearly 4 D after 48 h in D_2_O. These results are in good agreement with those of N-Acetyl-L-tyrosine as they nicely illustrate the fact that (i) at T0, i.e., just after irradiation, four dimeric structures are detected; (ii) among these four species, two (P_2_ and P_3_) disappear probably in favor of a fifth one (P_5_); (iii) cyclizations by Michael addition leading to final products are slow, more than several hours. It should be noticed that P_5_ is a broad chromatographic peak that could be composed of several species, such as diastereoisomers.

#### 2.1.3. Human Centrin 2

**Figure 3:**
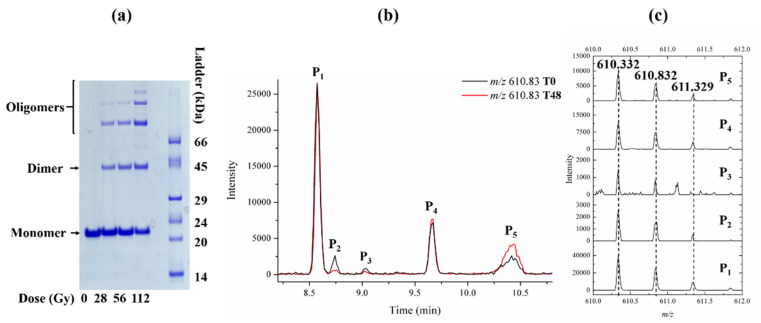
HsCen2 oligomerization at 40 Gy in N_3_^•^ production conditions. (**a**) SDS-PAGE to evidence covalent oligomers; (**b**) mass analysis of tryptic fragment before and after a 48 h incubation at 37 °C, extracted ion chromatograms (*m*/*z* 610.83 ± 0.05) corresponding to KTSLY-^C^YLSTK^N^ dimer; (**c**) full scan MS of dimer for [M + 2H]^2+^.

The application of this methodology to proteins is not straightforward. First, radiolytic oxidation leads to small dimer quantities (less than 10% of the initial protein concentration) regardless of dose. This is illustrated in Figure 3a, showing a 12% electrophoresis gel run under nonreductive conditions. The new bands appearing with the increasing irradiation doses correspond to covalent dimer, trimers and higher oligomers as first estimated with the molecular mass ladder and more precisely determined by mass spectrometry analysis [21,22]. Therefore, for higher doses, dimers are probably consumed to form trimers and higher molecular weight species. Second, to avoid a first separation step that could induce some modifications or loss of oxidative products, we decided to work on the global mixture obtained after irradiation, i.e., a sample predominantly containing protein monomers, some dimers in addition to oligomers. Furthermore, the separation step must be efficient enough to separate isomers. In order to achieve this, an enzymatic digestion step was necessary to obtain short peptides that could be separated by LC–MS in contrast to the whole protein. For human centrin 2, we performed tryptic cleavage at 37 °C for 4 h, which means that there is no T0 condition corresponding to samples frozen just after irradiation due to the evolution of the peptides during the peptide cleavage step. Nevertheless, to study the kinetics of the protein cyclization, we first incubated the irradiated protein at 37 °C for 48 h and then performed tryptic digestion and compared this condition with the protein digested just after irradiation. 

Chromatographic conditions to obtain a full protein sequence coverage and allow separation of five dimers were already finely tuned [14]. Just after digestion, extracted ion chromatogram at *m*/*z* 610.83 of KTSLY [M + 2H + 1]^2+^ shows the formation of five dimers (P_1_ to P_5_). The choice of the selected charge state is based on the fact that it gives the most intense signal, and to avoid any trimer contribution, we selected the mass [M + 2H + 1]^2+^. Incubation at 37 °C for 48 h reveals the decrease of P_2_ and P_3_ and the increase of P_5_. Based on the KTSLY dimers formed from pure peptide identified in the previous paragraph and analyzed under the same chromatographic conditions, we can conclude that the human centrin 2 dimerization process induces the formation of at least five dimers evolving to three in 48 h (the *ortho-ortho*, the *iso* dimer and one MAD) and we can suggest the formation of the same set of dimers based on their close retention times. Thus, even at the protein scale, several dimers are evidenced and evolve with time through Michael addition. The cyclization process occurs very slowly as the double cyclization needs more than 48 h to be completed as intermediate species (P_2_ and P_3_) are still visible at this time scale. We have here evidence that the cyclization process is slower for the full protein than for the isolated peptide. Thus, the presence of the full protein does not change the products but seems to change the cyclization kinetics.

### 2.2. Impact of the Carboxylic Function Modification

#### 2.2.1. N-Acetyl-L-Tyrosine Ethyl Ester (Ac-Y-Et)

**Figure 4:**
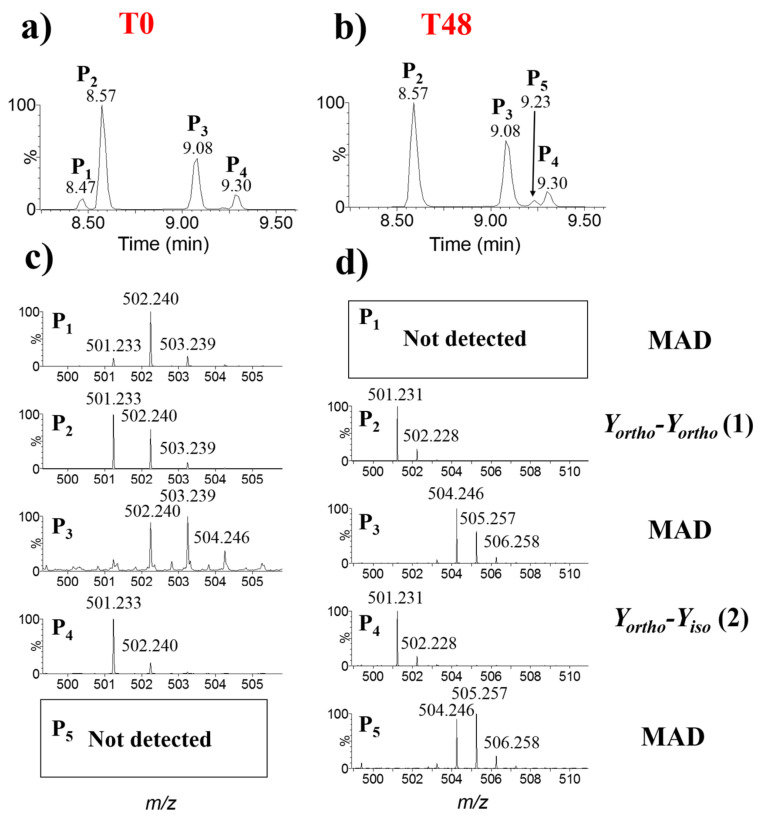
Oxidation of N-Acetyl-L-tyrosine ethyl ester (Ac-Y-Et) at 40 Gy in N_3_^•^ production conditions. Extracted ion chromatograms *m*/*z* 501.23 ± 0.05 [M + H]^+^ after oxidation in H_2_O at (**a**) T0 (0 h) and (**b**) T48 (48 h). Full scan MS spectra extracted at RT of dimers after oxidation in D_2_O at (**c**) T0 (0 h) and (**d**) T48 (48 h). Minor peaks in mass spectra arise from isotopic profiles.

Regarding the mechanism we proposed for the formation of final products, we considered that both terminal carboxylic and amine functions could be involved in the Michael addition cyclization. In the previous section, we focused on acetylated amino-acid, peptide and centrin 2 which, have in common the presence of a carboxylic acid group borne by the tyrosine residue. Nevertheless, in most of the proteins, the tyrosine residue will be incorporated within the core of the sequence. Therefore, we will turn now towards tyrosine containing compounds for which both the amino and carboxyl side are involved in an amide function of the peptidic bond. First, we considered a totally protected tyrosine with Ac-Y-Et. For this compound, tyrosine dimerization results at T0 in four peaks with retention time ranging from 8.5 to 9.5 min (P_1_, P_2_, P_3_ and P_4_ in Figure 4a) and finally four peaks after 48 h at 37 °C (P_2_, P_3_, P_4_ and P_5_ in Figure 4b), all corresponding to covalent dimers with monoisotopic mass at *m*/*z* 501.23 for the [M + H]^+^ species (Figure S2). In contrast with the previous examples shown above, Ac-Y-Et has a particular behavior as three peaks (P_2_, P_3_ and P_4_) detected at T0 are still present at T48. When irradiations were performed in D_2_O (Figure 4c,d), P_4_ mass is not affected by isotopic modification at T48. The P_2_ behavior is more complicated. At T0, *m*/*z* 501.23 is still the highest peak but its isotopic profile is not consistent with a pure species, as its contribution at *m*/*z* 502.24 is too high for a dimer, i.e., without incorporation of deuterium atom. We therefore consider that at this retention time, we have a second peak that evolves with time and disappears. After 48 h, P_2_ profile is coherent with a pure dimer insensitive to D_2_O, similarly to P_4_. Thus, we can attribute P_2_ to a co-elution of the *ortho-ortho* dimer and of a transient, D_2_O exchanging intermediate. P_4_ can be attributed to the *iso* form. For P_3_, H/D exchange is very interesting as it clearly illustrates a slow deuterium incorporation with a value of +2.7 D at T48 (Table S2). Thus, P_3_ results from a double cyclization that is formed very rapidly, as already detected at T0, but it exhibits a very slow exchange process with D_2_O. Based on their mass increase when irradiated in D_2_O, P_1_ detected only at T0 is interpreted as an intermediate MAD, and P_5_ corresponds to double cyclized dimer, since its isotopic profile is similar to that of P_3_ (Figure 4d and Table S2). P3 and P5 then correspond to **3a**, **3b** or **3c**.

These results are surprising as by protecting both the amino and carboxylic sides, we expected to prevent some cyclizations and thus to reduce the number of dimers. But in this case, the MAD family is still made of two compounds, the major one being formed very rapidly. Based on the reactivity of the functional groups of Ac-Y-Et, we propose that, as already evidenced [23,24], only the C=O amide function could be involved in a Michael addition and form 2 diastereoisomers.

#### 2.2.2. KTSLYG Peptide

**Figure 5:**
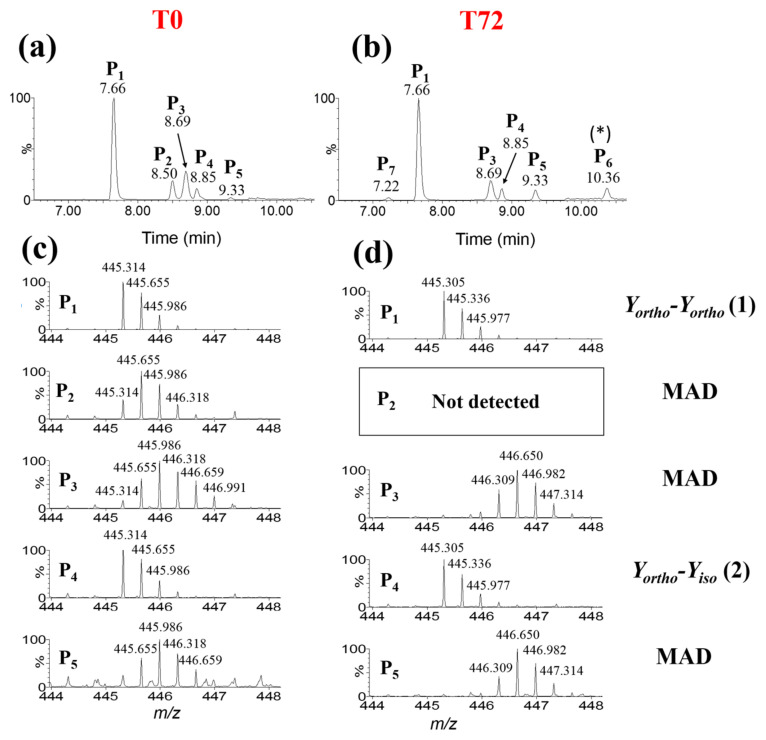
Oxidation of peptide KTSLYG at 40 Gy in the presence of N_3_^•^. Extracted ion chromatograms *m*/*z* 445.31 [M + 3H]^3+^ after oxidation in H_2_O at (**a**) T0 (0 h) and (**b**) T72 (72 h). Full scan MS spectra extracted at RT of dimers obtained in D_2_O at (**c**) T0 (0 h) and (**d**) T72 (72 h). (*) P6 intensity was multiplied by a factor of 10. Minor peaks in mass spectra arise from isotopic profiles.

To confirm that double addition can occur whatever the tyrosine localization in the sequence, we investigated a 6-residue peptide (KTSLYG) without C-terminal tyrosine. Figure 5 illustrates the diversity of ion extracted at *m*/*z* 445.31 corresponding to the [M + 3H]^3+^. Just after irradiation (T0), five major peaks (P_1_ to P_5_) are detected though after 72 h, P_3_ is no more detectable but P_6_ and P_7_ now appear (Figure 5a,b). Full mass spectrum of each peak confirms the covalent dimerization of peptide KTSLYG after irradiation and the fragmentation pattern is in agreement with a covalent bond formed at the tyrosine residue (Figure S2). Irradiation in D_2_O solution reveals that two species are not sensitive to H/D exchange with the solvent, which leads us to conclude that P_1_ (RT = 7.66 min) is the *ortho-ortho* dimer and P_4_ (RT= 8.85 min) is the *iso* dimer. As already illustrated with Ac-Y-Et, P_3_ is present both at T0 and T72 and exhibits a slow deuterium incorporation with time. At T72, its mass variation (+4 D) confirms that it is formed very rapidly after a double cyclization. From its isotopic profile at T0, P_2_ is identified as an intermediate MAD with only one cyclization. P_6_ and P_7_ are clearly detected at 72 h in H_2_O, but the low signal intensity in D_2_O makes it difficult to precisely determine their mass increase. This peptide dimerization behavior, very similar to that of Ac-Y-Et, confirms that even when implicated in peptidic bonds, CO amide functions can be implicated in Michael additions. Considering that two amide functions can be close to the Tyr residue, the MAD family could be made of numerous isomers, in agreement with the presence of P_6_ and P_7_. 

#### 2.2.3. Human Calmodulin

**Figure 6:**
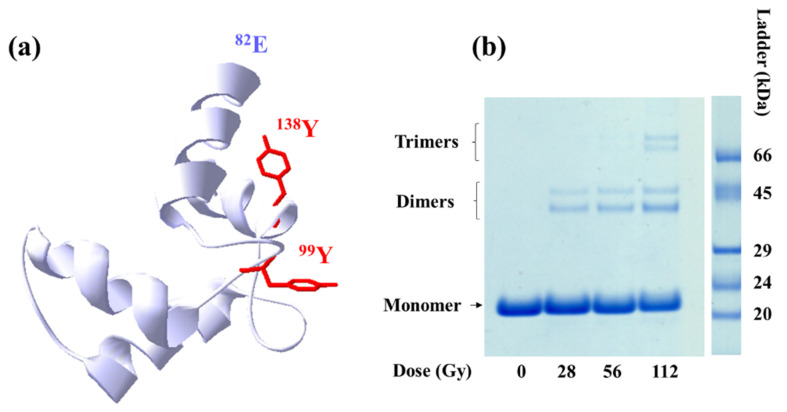
CaM oligomerization at 40 Gy in the presence of N_3_^•^. (**a**) Local representation of Tyr residues of CaM (from [25], 3cln PDB structure) and (**b**) SDS-PAGE of CaM after radiolytic oxidation by N_3_^•^ for doses up to 112 Gy.

To transpose this study to a more biological context and to explore the impact of tyrosine localization in a secondary structure, we decided to study calmodulin dimerization when submitted to azide radicals. Calmodulin possesses two tyrosine residues, ^99^Y located in a turn and ^138^Y in helix VII [25,26] as illustrated in Figure 6a. Irradiation of CaM in the presence of azide radicals reveals in SDS-PAGE the formation of dimers as can be estimated from the position of new bands increasing with the dose (Figure 6b). Interestingly, two different species with a molecular weight corresponding approximatively to dimers are evidenced. This difference in migration is not so surprising. Though the protein is denatured by heating prior to migration and the presence of SDS, two different dimer conformations could behave differently during electrophoretic migration [27].

**Figure 7:**
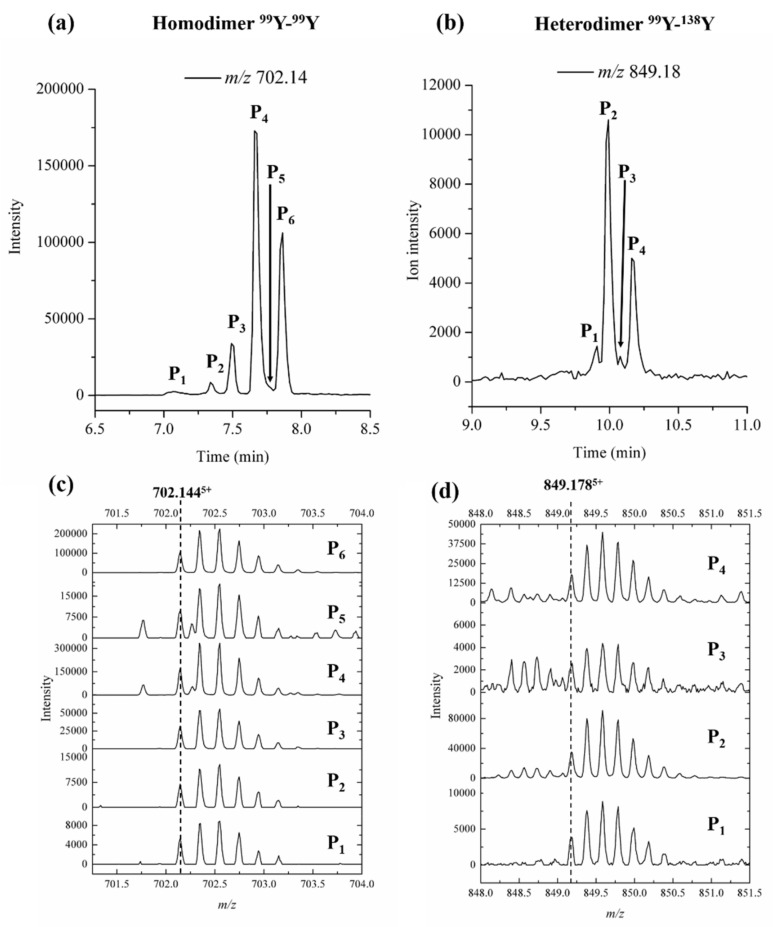
Tryptic digestion of oxidized CaM at 40 Gy in N_3_^•^ production conditions. Extracted ion chromatograms (*m*/*z* 702.14 ± 0.05, [M + 5H]^5+^) for (**a**) the homodimer ^99^Y-^99^Y in fragment ^92^V-R^107^ and (**b**) (*m*/*z* 849.18 ± 0.05, [M + 5H]^5+^) for the heterodimer ^99^Y-^138^Y. Full scan MS spectra extracted at RT of dimers for (**c**) ^99^Y-^99^Y, (**d**) ^99^Y-^138^Y.

For CaM, tryptic digestion was performed after 4 h at 37 °C without any previous incubation at 37 °C. From UPLC-MS experiments, we were able to obtain a 100% protein coverage after enzymatic cleavage. Tyrosine residues are now found in different peptidic fragments but mainly ^99^Y in fragment ^92^V-R^107^ at RT = 7.02 min and ^138^Y in fragment ^128^E–K^149^ at RT = 11.58 min. As radical oxidation by N_3_^●^ only generates Tyr-Tyr dimers we can expect three combinations between CaM Tyr residues: two homodimers ^99^Y-^99^Y and ^138^Y-^138^Y and one heterodimer ^99^Y-^138^Y. Among these three possibilities, we only evidenced on homodimer and one heterodimer. First, dimeric fragments were detected from 7 to 8 min on the ion extracted at *m*/*z* 702.14 for [M + 5H]^5+^ corresponding to the homodimer of ^99^Y in fragment ^92^V-^107^R (Figure 7a). From Figure 7a, it is clear that at least six different peaks respond to this mass, confirmed by their full mass spectra (Figure 7c). Second, from RT between 9.5 to 10 min for *m*/*z* 849.18 [M + 5H]^5+^ correspond to a hetero-dimer ^99^Y–^138^Y (Figure 7b,d). Here, we were able to separate up to four dimers. Interestingly, we were unable to identify any homodimer of ^138^Y for the fragment ^128^E-^149^K. Nevertheless, these results clearly illustrate the fact that even for tyrosine residue contained in a peptidic sequence and more precisely here for ^99^Tyr in an alpha helix, the dimerization process is as diverse as for simpler or not structured systems.

## 3. Discussion

In this study, we refined our knowledge of tyrosine dimers variety that can be formed in proteins after oxidative attack. From simple systems as amino acids to peptide and finally proteins, we confirmed that three initially formed cross-links between tyrosine residues can lead to many more final species. In addition to *ortho-ortho* and *iso* dimers already known, the MAD family is formed arising from Michael additions and thus two successive cyclizations. In the literature, such cyclization is often evoked with a catalysis by amine functions. In proteins, residues close to the tyrosine cross-link, such as lysine, could favor this addition, as it was already proposed [28,29]. Specific protected forms of tyrosine amino acid highlighted the fact that such additions are also possible via amide functions and thus they can occur on both sides of a Tyr residue in proteins. Then, considering the structures proposed, it is obvious that many more dimeric forms exist than those drawn in Scheme 1. One should also consider that in the MAD family, additional asymmetric centers are formed upon cyclization. Therefore, we can expect to have more dimers than those that were able to separate by the formation of diastereoisomers. This is what is suggested by the several small peaks detected in the case of the KTSLYG peptide and calmodulin, by the drift of the baseline already evoked for tyrosine [15], that could account for many different species, and also by the width of P_5_ for the peptide KTSLY and human centrin 2. So, improvement of dimer separation is questionable. We performed all the separations on a UPLC system equipped with a C18 column, achieving a very high resolution. Nevertheless, a separation based on chirality or with another type of stationary phase might improve dimers enumeration. Moreover, though more diastereosiomers than what we detected can be expected, they probably do not all correspond to favored structures. Shamovsky et al. [30] confirmed the stability of some proposed reaction intermediates and the structure of the *ortho-ortho* and *iso* Tyr dimers with DFT and ab initio methods on cresol. Hence, such theoretical study on the MAD family should be an important complement to our experimental study and greatly enhance our knowledge of existing dimer structures. Furthermore, the impact that the protein structure and/or sequence could have on the number of dimers needs further studies. Indeed, we focused on two proteins that have accessible tyrosine residues. Human centrin 2 possesses only one tyrosine, which is the C-terminal residue, in a very flexible region. Therefore, structural rearrangement after Michael addition is not expected to be much perturbed by the secondary structure of the protein. For human calmodulin, one Tyr residue is located in a turn and the second one in an alpha helix. As already showed for UV radiation [31], only two cross-links were evidenced, between residues ^99^Y-^99^Y and ^99^Y-^138^Y. But for both of them, many different dimer isomers were evidenced. Though this cannot be generalized to any protein structure, it clearly shows that even between two helices, a tyrosine dimer can evolve by Michael addition to generate more constrained structures. As protein structure modifications and decreases of enzymatic activity were often evidenced after oxidative damages [32], we can wonder whether the MAD family formation could be linked to such phenomena.

Furthermore, in this study we only focused on qualitative characterization of the dimers. With mass spectrometry as a detector, correlating peak intensity with species quantity is not straightforward as relative intensities also reflect molecule ionization efficiencies and not only their respective proportions. UV detection was also performed in this study; nevertheless, some dimers were hardly detected, which is not so surprising as some of them lose their aromaticity and therefore a specific chromophore. For quantification, it would also be necessary to determine each species absorption coefficient. Without commercial standards and considering the synthesis complexity for all the new dimers, it is not possible for the moment to consider their relative proportions. Nevertheless, taking into account the role they could play in vivo, we consider as mandatory now to develop new approaches to be able to quantify all these species in vitro but also in vivo. 

For the present work, radicals were produced by gamma irradiation of aqueous solutions based on radical production by water radiolysis. More precisely, we adapted radiolytic conditions (N_3_^●^ versus HO^●^) to only consider dimerization among all oxidative damages. Nevertheless, radical can be produced by many other physical processes such as photolysis, chemical reactions as Fenton reaction or biochemical reactions. Moreover, it has already been well illustrated that tyrosine radicals can be formed in proteins after electron transfer from Trp residues [33,34,35]. Thus, whatever the way TyrO^•^ is produced, oxidation leading to tyrosine dimers is expected in vivo and all the different isomers identified here are expected to be formed. This raises the question of the impact they could have in vivo. Moreover, other important information obtained from our results is that evolution of *ortho-para* tyrosine cross-link through Michael addition can be a slow process, up to 72 h at 37 °C. This means that both the intermediates and the final products could be found in vivo for a non-negligible amount of time. Additionally, as MAD are quite reactive species, they could be involved in intermolecular processes in addition to the unimolecular processes discussed in this article. 

As already stated, highlighting new tyrosine dimers and their structure characterization are of great importance as di-tyrosine is already employed as a biomarker of oxidative pathologies [11]. Indeed, di-tyrosine has been notably found in brain proteins from Alzheimer’s patients [36], in human atherosclerotic plaques [37] or in the urine of people with diabetes [38]. Current protein di-tyrosine detection is based either on fluorescence or on antibody signaling. Nevertheless, we already showed that only the ortho-ortho dimer is fluorescent [15]. Furthermore, considering the constrained structures of the MAD family, we can wonder whether antibodies recognition is still efficient. As a consequence, these new tyrosine dimer structures should be very helpful in designing more accurate probes. Considering di-tyrosine structure diversity, including the long-lived intermediates species, and the absence of knowledge of their specific proportions, we can raise the question of the role or toxicity of all these dimers in vivo.

## 4. Materials and Methods

### 4.1. Amino Acids and Peptides Preparation

All sample solution were prepared in ultrapure water (18 MΩ.cm) at a concentration of 50 µM in phosphate buffer 10 mM pH 7.4. For D_2_O experiments (99.8% purity, Acros Organic, Illkirch, France), the procedure was the same as for H_2_O but D_2_O was used instead.

Peptides were provided by Proteomic Solutions (Saint Marcel, France), with a purity >98%.

### 4.2. Protein Production and Purification 

#### 4.2.1. Human Centrin 2

Human centrin 2 was produced in E. Coli and purified as already described in Blouquit et al. [21]. The protein sequence is given below:

MASNFKKANMASSSQRKRMSPKPELTEEQKQEIREAFDLFDADGTGTIDV

KELKVAMRALGFEPKKEEIKKMISEIDKEGTGKMNFGDFLTVMTQKMSEK

DTKEEILKAFKLFDDDETGKISFKNLKRVAKELGENLTDEELQEMIDEADR

DGDGEVSEQEFLRIMKKTSL**Y**

#### 4.2.2. Human Calmodulin 

Human calmodulin production was performed as in [39]. For purification, cells were disrupted in a glycerophosphate buffer (50 mM), pH 7.4, containing EDTA (2 mM), NaCl (50 mM), β-mercaptoethanol (2 mM), and an antiprotease cocktail (Complete, Roche, Basel, Switzerland), using a 2 kbar cell disrupter (Constant Systems, Warwick, UK). After 30 min centrifugation at 20,000× *g*, the supernatant was loaded on a DEAE-TSK column, equilibrated with a similar glycerophosphate buffer at pH 8.5, and eluted with a NaCl gradient from 0.05 to 0.25 M. The fractions containing CaM were then deposited on a phenyl-TSK column, equilibrated in glycerophosphate buffer (50 mM), NaCl (0.1 M), EDTA (5 mM) and pH 7.0 that was eluted with ammonium sulfate buffer from 2 to 0 M. The purified samples were concentrated on YM10 Diaflo ultrafiltration membranes (Amicon, Beverly, MA, USA) and desalted on a Sephadex G-25 column equilibrated with NH_4_HCO_3_ buffer (1.5%). Other chemicals were from VWR (Fontenay sous Bois, France) The CaM sequence is given below:

MADQLTEEQIAEFKEAFSLFDKDGDGTITTKELGTVMRSLGQNPTEAELQDMI

NEVDADGNGTIDFPEFLTMMARKMKDTDSEEEIREAFRVFDKDGNG**Y**ISAAE

LRHVMTNLGEKLTDEEVDEMIREADIDGDGQVN**Y**EEFVQMMTAK

### 4.3. Gamma Irradiation

Azide radical production results from water radiolysis were submitted to ~1.17 MeV gamma photons (^60^Co). After excitation or ionization of water according to Equations (1) and (2), solvated electrons and hydroxyl radicals were the main radicals formed [40]. By degassing all the solutions for at least 45 min with N_2_O, electrons were scavenged and transformed in HO^●^ radicals according to Equation (1). The presence of azide (NaN_3_) in all solutions allowed HO^●^ scavenging leading to only N_3_^•^ (Equation (2)). The dose rate was fixed at 10 Gy.min^−1^, 1 Gy corresponding here to 0.55 µmol of N_3_^•^.

(1) $$N_{2}O+{\;e}_{\mathrm{aq}}^{-}+H_{2}O\;\to {\;\mathrm{HO}}^{•}+{\mathrm{HO}}^{-}+N_{2}$$

(2) $$N_{3}^{-}+{\mathrm{HO}}^{•}\;\to {\;N}_{3}^{•}+{\;\mathrm{HO}}^{-}$$

After irradiation, samples were either frozen directly to −20 °C (T0) or incubated at 37 °C for evolution before freezing. In that case, time indicates the number of hours of incubation, for example, T48 for 48 h. 

### 4.4. Gel SDS-PAGE

SDS-PAGE electrophoresis was performed using a 12% acrylamide-bisacrylamide gel with a Tris-Tricine buffer [41] under non reductive conditions. Gels were running at 120 V for 4 h. Staining was performed overnight with Coomassie Blue R-250.

### 4.5. Analysis: UPLC–MS

UPLC–MS analysis were performed on the same system as in Gatin et al. [14]. The column and the mass parameters are identical, though specific chromatographic conditions were applied to each sample. Eluent composition and gradient elution is given for each sample. For KTSLY and digested proteins, the mobile phase eluents used were (A) 0.1% formic acid (*v/v*) in water and (B) 0.1% formic acid 90% acetonitrile (*v/v/v*) in water. For Ac-Y, Ac-Y-Et and KTSLYG, the mobile phase eluents used were (A) 0.2% formic acid (*v/v*) in water and (B) 0.2% formic acid 90% acetonitrile (*v/v/v*) in water. Gradient elution for Ac-Y is 10% B for 1 min, 10–35% B for 9 min. For Ac-Y-Et, the gradient was 0% B for 1 min, then 0–70% B for 14 min. Gradients for KTSLY, KTSLYG and digested human centrin 2 were 0% B for 1 min, then 0–15% B for 12 min. Finally, for human calmodulin, gradient elution is 0% B for 2 min, 0–12% B for 4 min and 12–50% B for 6 min. Column temperature was fixed at 30 °C and flow rate at 0.5 mL min^−1^ for each experiment except for KTSLY for which flow rate was 0.6 mL min^−1^.

For MS analysis, experiments were performed with an ESI (ElectroSpray Ionization) source in positive mode on a Q-ToF instrument from Waters Company (Manchester, UK) (SYNAPT G2-Si HDMS). Calibration using sodium trifluoroacetate was performed between *m*/*z* 159.016 and *m*/*z* 1926.317. Capillary voltage was set to 1.2 kV and desolvation temperature to 450 °C. Tolerance for data interpretation was ±50 ppm as no internal calibration was used during these experiments.

### 4.6. Data Treatment: Deuterium Average Incorporation

In order to estimate the average number of incorporated deuterium atoms during H/D exchange experiments, intensities of each contributors of the isotopic profiles were reported as well as their precise experimental *m*/*z*. Thus, a mass average weighted by the intensities was calculated when irradiated in both H_2_O and D_2_O. The mass average obtained in H_2_O is finally subtracted to the D_2_O one, to give the average number of added deuterium atoms. This average number is then multiplied by the charge state.

## Supplementary Materials

The following supporting information can be downloaded at: https://www.mdpi.com/article/10.3390/ijms23031174/s1.
