# Supplementary material for: Protein Dimerization via Tyr Residues: Highlight of a Slow Process with Co-Existence of Numerous Intermediates and Final Products
Source: Int J Mol Sci. 2022 Jan 21;23(3):1174. doi: 10.3390/ijms23031174 (PMC8835203; doi:10.3390/ijms23031174)
Supplement: Supplementary file 1 [file ijms-23-01174-s001.zip › ijms-1537471-supplementary.pdf]

International Journal of
Molecular Sciences

Supplementary Materials

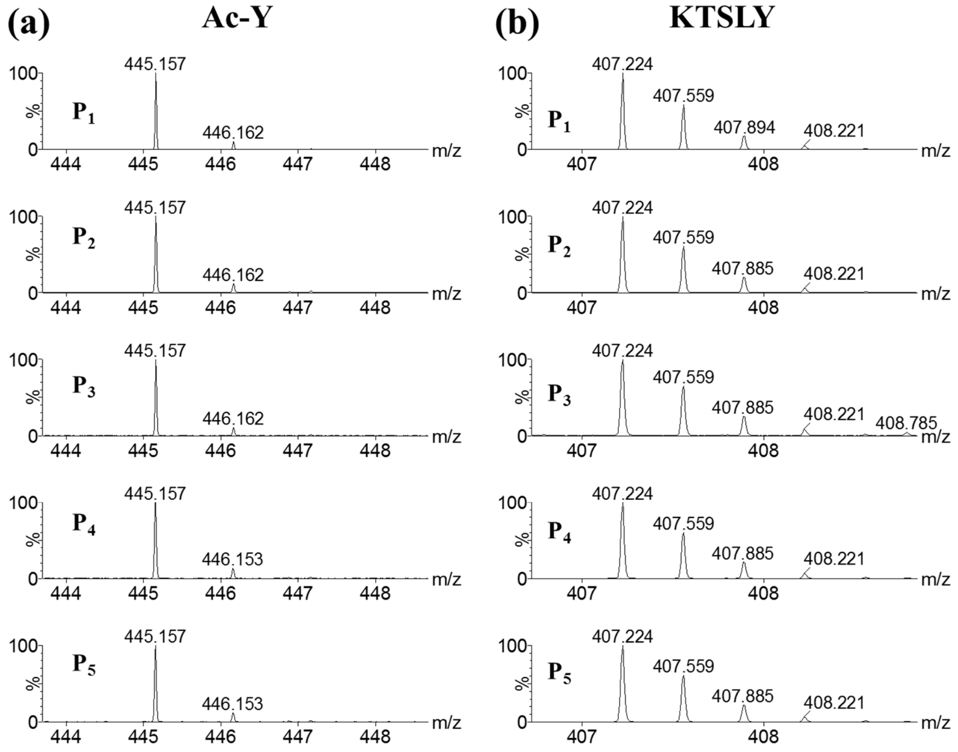

Figure S1: Oxidation of Ac-Y (a) and KTSLY peptide (b) in H_2O. Full scan MS spectra extracted at RT of dimers.

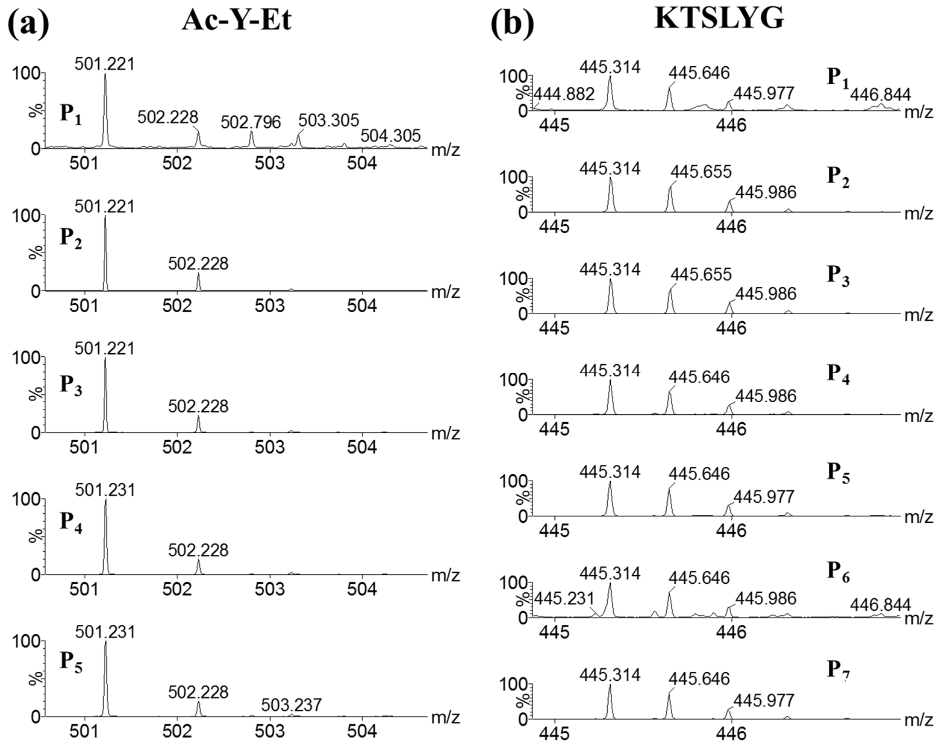

Figure S2: Oxidation of Ac-Y-Et (a) and KTSLYG peptide (b) in H_2O. Full scan MS spectra extracted at RT of dimers.

Monoisotopic *m/z* / Species	Monomer *(theoretical)*	Dimer $[M + xH]^{x+}_{dimer} = \frac{2 * M_{monomer} - 2 * M(H) + x * M(H)}{x}$		
		**[M+H]$^{+}$** *(theoretical)*	**Selected [M+xH]$^{x+}$** *(theoretical)*	**Detected [M+xH]$^{x+}$** *(experimental)*
**Ac-Y**	223.085	445.161^{+}	445.161^{+}	445.157^{+}
**Ac-Y-Et**	251.116	501.224^{+}	501.224^{+}	501.223^{+}
**KTSLY peptide**	611.341	1219.657^{+}	407.224^{3+}	407.235^{3+}
**KTSLYG peptide**	668.362	1333.700^{+}	445.238^{3+}	445.314^{3+}
**^{168}KTSLY^{172} in CEN2**	611.341	1219.657^{+}	610.332^{2+}<br>[M+2H+1] 610.836^{2+}	610.336^{2+}<br>[M+2H+1] 610.832^{2+}
**^{92}V-R^{107} in CaM**	1753.864	3506.719^{+}	702.150^{5+}	702.144^{5+}
**^{92}V-R^{107} in CaM**<br>**^{128}E-K^{149} in CaM**	1753.864<br>2489.073	4241.928^{+}	849.191^{5+}	849.178^{5+}

Table S1: Theoretical and experimental *m/z* of considered species.

		P_1	P_2	P_3	P_4	P_5	P_6	P_7
**Ac-Y**	T0	0.03	0.70	0.94	-0.01	ND	-	-
	T48	-0.01	ND	ND	-0.02	**2.24**	-	-
**KTSLY**	T0	0.09	0.87	0.75	0.09	1.35	-	-
	T5	0.05	1.59	1.64	0.07	**3.26**	-	-
	T48	0.10	ND	ND	0.07	**3.85**	-	-
**Ac-Y-Et**	T0	0.86	0.39	1.56	0.01	-0.04	-	-
	T48	ND	-0.03	**2.70**	0.01	**2.66**	-	-
**KTSLYG**	T0	0.06	0.70	1.83	0.14	1.44	ND	ND
	T72	0.01	ND	**3.49**	0.01	**3.53**	**3.44**	**2.93**

Table S2: Average deuterium incorporation after H/D exchange for the different chromatographic peaks.
